# Surgical patients’ perspectives on nurses’ education on post-operative care and follow up in Northern Ghana

**DOI:** 10.1186/s12912-018-0299-6

**Published:** 2018-07-09

**Authors:** Bernard Atinyagrika Adugbire, Lydia Aziato

**Affiliations:** 1 Nurses’ Training College, Zuarungu, Ghana; 2 Department of Adult Health, School of Nursing, College of Health Sciences, University of Ghana, P.O. Box LG 43, Legon, Accra, Ghana

**Keywords:** Discharge planning, Home care, Patients’ education, Patients’ experiences, Personal hygiene, Wound care

## Abstract

**Background:**

The purpose of the study was to explore surgical patients’ experiences of discharge planning and home care in the Northern part of Ghana.

**Methods:**

The study was conducted at a referral hospital located at the Northern part of Ghana. A qualitative explorative descriptive design was adopted for the study. Purposive sampling technique was used to recruit participants. Data was saturated with 15 participants aged between 23 and 65 years. All the interviews were audio-taped and transcribed verbatim. Data analysis was done using the processes of content analysis.

**Results:**

Nurses educated surgical patients on discharge to avoid smoking, alcohol drinking, chewing cola nuts and strenuous exercise to promote healing and prevent complications. Patients were educated to keep their wound dry and clean. Patients were advised to eat nutritious food, vegetables and fruits and take their medications as prescribed. They were to report drug effects and come to the hospital for follow-up visits. Patients were urged to come for daily wound dressing at the outpatient department. On the contrary, some nurses did not educate patients on signs of wound healing or infection. Some nurses were rude to the patients during wound dressing. Nurses did not visit patients at home when they were discharged from the hospital.

**Conclusions:**

The study showed that although nurses were able to educate discharged patients on how to manage their health at home, there is the need to improve communication and attitude to enhance care.

## Background

Early post-operative recovery depends on educating patients on self-care, wound care and providing clear information to discharged patients [1, 2].Early post-operative recovery also depends largely on good interactions between the hospital nurses and the community health nurse to ensure smooth handing over of patients to continue treatment especially patients’ wound care. Bodily care includes looking after oneself, eating well and exercising regularly through proper teaching. In a study conducted in Northern Taiwan,the results indicated that to ensure smoking cessation in post-discharged patients, effective cessation counselling including encouraging patients to modify their lifestyle through effective education is helpful [3].Ineffective teaching following discharge may lead to the patients’ lack of knowledge about how to care for self at home and become ignorant of signs and symptoms of impending infections or complications [4, 5].In clinical practice, it is observed that hospital discharge instructions are given at the moment patients are about to leave the hospital instead of being developed throughout the hospitalization period. As a result the patient leaves the hospital with incomplete information about care including instructions for self-care at home [6].

Post-operative wound care is an important part of self-care after discharge. However, it has been stated that discharged surgical patients lack the required knowledge for wound monitoring. This is attributed to poor discharge teaching, lack of self-efficacy for wound care at home and inaccessible communication with nurses about wound; resulting in wound infection [7].This corroborates anecdotal evidence at the Regional Hospital, Bolgatanga, Ghana where many of the surgical patients wounds are often infected due to failure of some nurses to educate patients during discharge planning and to properly hand over discharged patients to community health nurse to continue wound dressing.Also it was reported that more than 85% of surgical patients usually rely on family members or friends for support especially with wound care, household activities, and mobility [8]. This demands inclusion of the family in post-operative wound care education. However, the current study recruited only patients as they were the population target of the researcher and were also readily available, Future researchers could consider researching on family education.

Also, the literature revealed that some nurses fail to explain the purpose and side effect of medications to some patients when they are discharged. Those patients that received information on medications such as drug actions and side effects felt that the information was not clear [9, 10]. However, some patients have adequate discharge instructions on their medications including timing and route of administration. Exercise and nutritious diet were also emphasized on discharge [8]. For instance, some discharged patients received tailored discharge instruction on medications, wound dressing and prevention of infection. In a similar study, the results indicated that 72% of patients receive discharge instruction from other health personnel instead of nurses [11].

In some of the studies, patients whose conditions improved during admission were discharged without any guidelines or instructions from nurses. Patients were not accompanied out of the building neither where their departure needs provided. In addition, there was no time a nurse assessed their learning needs, gave them advice on resources or enquired about their home situation with regard to their home care that could help limit their care burden [12, 13] These studies have shown that some nursing staff never planned for the patient discharged and this may compound patients post-operative complications such as infection.

In a survey conducted on patients with complex care needs in 11 countries (Australia, Canada, France, Germany, the Netherlands, New Zealand, Norway, Sweden, Switzerland, the United Kingdom, and the United States), it was reported that one in four patients did not receive instructions for follow-up nor did they receive clear medication directions [14]. Also, there is untimely, infrequent follow up when patients are discharged making it difficult for patients to manage their conditions at home leading to surgical site infection after being discharged. This puts much burden on patients at home who are not well prepared to manage it resulting in readmission [7, 15, 16]. As a result, surgical patients believed that mobile health monitoring is highly acceptable. Nurses need to provide more frequent, thorough and convenient follow-up to assess their state of health at home upon discharge.This could reduce post-discharge anxiety and help minimize the risk of wound infection and adverse drug effect after hospitalization [7, 17].In a similar study, it was also reported that patients with long term conditions were referred to community services for ongoing support [18]. Comparatively, some of these literatures go to confirm the practice of nursing care at the regional hospital. In Ghana, the training and regulatory body of nursing (Nursing and Midwifery Council of Ghana) curriculum demands that the training of registered general nursing programme should equip qualified nurses the necessary kills to effectively hand discharge patients over to public health nurse or community health nurse at the community for continuity of care. As a result, during training, student’s nurses are mandated to write a project work on individual patient care study. This project allows the students to admit and nurse the patient till he/she is discharged. They visit the homes of these patients to assess the patient home environment whilst the patient is on admission in order to prepare the patient well for discharge. They also visit the patient at home upon discharged to assess and see whether the patient is implementing the education. They finally terminate the care by handing the patient over to the community health nurse for continuity of care. However, anecdotal evidence showed that qualified nurses do not perform these functions despite there are no hospital policy restrictions, hence compounding discharged patients problems. Rather, these discharged patients are often referred to the public health unit even though nurses are expected to make follow up at the community level to assess the patient condition and provide necessary education for speedy recovery.

With perioperative care, patients’ perspectives on discharge is important since many outcomes such as health related quality of life which includes the desire to regain health and satisfaction of care can only be reported by the patients [19, 20]. This has been confirmed at Kenyatta National Hospital where 167 participants representing 52.4% admitted their satisfaction with nurses’ education on their wound dressing when they were discharged [21]. In addition, surgical patients prefer wound dressing materials that promote quick wound healing, reduce pain and ensure shortest hospitalization time [22]. However, at the surgical unit of the regional hospital, there are no specific wood dressing materials that nurses’ use apart from the ordinary cotton wool and gauze provided. Besides, despite the fact that each participant went through different surgical procedure, the nurses use the same dressing materials for wound dressing. However, the frequency of wound dressing and the dressing lotion changes –thus as part of the hospital local practices, non infected wounds are being dressed with methylated spirit using alternate days whilst infected wounds are dressed with povidone iodine. Also, when there is need for wound debridement as part of the hospital policy, the patient is readmitted and sent to theatre for wound toileting. Hence, there is the need to educate discharged patients on the kind of wound dressing materials to use at home that will enhance speedy wound healing.

The curriculum use for training nurses in Ghana demands that student nurses be taught effective therapeutic communication skills to enhance good interpersonal interactions with patients at the wards upon completion of their training. They have also been taught on how to prepare a patient for discharge. However, these acquired skills are abandoned by nurses during their duties. This therefore confirms studies that have observed that basic training of nurses on communication skills; regular in-service training and workshops are recommended as ways that could improve on the quality of nursing care especially with discharge preparation [23, 24].Besides, it is important to ensure effective nurse-patient interaction so that nurses would be able to explain the specific disease of the patient [25, 26]. However, some patients claimed nurses spent little time with them and were always in a hurry and busy and as a result communicated poorly with them. This gave them the impression that they lacked time to talk to, listen or be with them [27, 28].

In summary, the literature has indicated that there are gaps with regard to discharged patients’ home management of health conditions. The study therefore explored the experiences that discharged surgical patients from the Northern part of Ghana encounter during management of their health condition at home. It examined the techniques and skills that nurse imparted into them during discharge period to enable them care for themselves at home. The literature also examines the interactions that patients encounter at the community level especially, with the handing over of the patients to the community health nurse to continue treatment. The information gathered through this exploration would inform various stakeholders and hospital management including ward managers about the challenges that these discharged patients go through at home.

## Method

### Design

The study adopted exploratory and descriptive qualitative approach to establish a comprehensive insight into the patients’ experiences of discharge and home care provided by nurses. The design allows an exploration of participants’ feelings, behaviour, thoughts, insight and action [29].

### Setting

Ghana is located in the Western African region, surrounded by the Gulf of Guinea to the South. The country is formed from the union of the Gold Coast, Ashanti Protectorate, Northern Territories and British Togoland. Ghana is slightly smaller in size compared to Oregon. This tropical sub-Saharan nation encompasses approximately 92,000 mile^2^ of territory, ranking as the 82nd largest country in the world. Ghana shares around 1500 miles of its land borders with its nearest neighbours, that is, Burkina Faso to the North, Cote d’Ivoire to the West and Togo to the East.

The Upper East Region is located in the northern part of Ghana and is the second smallest of ten (10) administrative regions in Ghana, occupying a total land surface of 8842 km^2^ or 2.7% of the total land area of Ghana. The Upper East regional capital is Bolgatanga where the regional hospital is located. The major towns in the region include Navrongo, Paga, Bawku and Zebilla. Comparatively, the region has varied population comprising all manners of tribes with different spoken languages. However, the commonest languages that are spoken are Grunne (local language) and English language.

The study was conducted at the Regional Hospital located at the Northern part of Ghana. The hospital is located at the North-Eastern part of the regional capital, Bolgatanga. The regional hospital is the largest hospital in the region; as a result, it serves as a referral centre for the district hospitals in the region. The entire nursing population of the hospital is about 160 and the total bed capacity is two hundred and six (206) beds. The surgical department is attached to the theatre and about 50 surgeries are performed in a day. Comparing the setting to other settings cited in the study especially the Europeans countries, it is obvious that the study setting is small and does not have the requested logistics to meet the modern standard of care expected. Even though the nurses are trained to provide standardised care, the nature of the setting restricts the kind of care to be provided. Many at time nurses do improvise certain materials to provide certain nursing care to patients. For instance, making a follow up upon the discharge of a patient is often difficult because of lack of means as compare to other settings where means of transport is not a challenge. Also, the hospital telephone network system is poor hence nurses find it difficult to make a telephone call to patients upon discharge. Nurses can only do this by using their cell phones.

### Population and sampling technique

The study population was discharged surgical patients who went through either emergency or planned general surgical procedures within 1 month and stayed within Bolgatanga Municipality. The study involved both males and females general surgical patients who came to the surgical unit as outpatients for their daily wound dressing. The study adopted purposive sampling technique to collect the data. Purposive sampling technique was employed because of the identifiable nature of participants. That is, the study population was participants who have had general surgeries and had been discharged within 1 month. Hence, 15 participants were recruited based on data saturation. The surgical records of the participants at the hospital were used to identify the participants especially records at the male and female surgical wards and the theatre. The participants were also contacted on phone.

### Data collection tool and procedure

A semi-structured interview guide was used to conduct face to face interview with the participants. The lead author conducted all the individual interviews after he had acquired the necessary skills through training to conduct qualitative interviews. The interviews were conducted in English and Grunne (local language). Probes were used to generate detailed understanding of participants’ experiences of home care and nurses’ follow- ups following participants discharged from the ward. The interviews were conducted at a time and place convenient to the participants and lasted between 30 to 45 min. The same interview guide was used throughout for all the participants that were involved in the study. These interviews were continued till the 15th participant, when the researcher realised that all the15 participants were giving similar responses indicating that there will not be any new information to be provided by the participants even if the researcher wishes to continue with the interviews. Hence, base on this, it was an indication that the data were saturated. The interview was also recorded and transcribed in English. Field notes on observations made during the interviews including non-verbal cues of the participants were written during transcription to reflect participants’ true experiences of home management and follow-ups by nurses.

### Data analysis

The principles of thematic content analysis according to [30] were used to analyse data. The data were analysed concurrently with data collection to allow for the search of important themes and patterns in the data. The authors coded the data independently and the discrepancies were discussed for a consensus during the analysis. The data were organized into themes and sub-themes after making meaning of the transcripts. The authors discussed the themes and sub-themes to ensure the true reflections of participants home management experiences and nurses’ follow-ups. The authors managed the data manually to extract data that supported the findings.

### Rigour

Rigour was ensured through member checking where participants confirmed their experiences during data analysis. There was team discussion of emerging themes to facilitate an in-depth understanding of the participants’ experiences of home management and nurses follow ups. Also to ensure that the findings can be applied to other settings, we provided a detailed description of the research processes and findings. The same semi-structured interview guide was used to ensure consistency of the data collection.

## Results

### Demographic characteristics

A total number of 15 participants were interviewed during the research. Eight (8) participants were females and seven were males. These participants were aged between 23 and 65 years. Three of the participants were in their early twenties, five were in their mid-thirties, four were in their mid-fifties, two participants were in their late fifties and one participant being exactly 60 years. Nine (9) of these participants had some sort of formal education and could therefore speak English fluently. The remaining six (6) participants did not have any form of education and therefore could speak only Grunne. Eleven of the participants comprising five (5) males and six (6) females were married while three males and a female were not married.Three of the participants were Muslims, eight were Christians while four (4) were traditionalist. In terms of occupations, eight of these participants were trained teachers, two were motor fitters and the remaining five participants were peasant farmers. All the fifteen (15) participants were resident within Bolgatanga municipality.

Two major themes were identified in the study. These themes included patients’ discharge information and nurses’ follow ups/ home visits.The sub-themes of patients’ discharge information included information on nutrition and lifestyle practices, medications and wound care and nurse- patient interactions.

### Patients’ discharge information

Participants’ experiences on discharge information centered on the education that participants received from nurses before they were discharged to help them care for themselves at home. Participants received educationthat was beneficial to their healthcare at home on areas such as lifestyle modifications, good nutrition, medication and wound care.

### Nutrition and lifestyle modifications

All the participants reported that nurses told them that they should not smoke or drink alcohol such as a locally brewed alcoholic beverage (pito) as it could affect wound healing.

*“The nurses told me that I should not drink pito or smoke cigarette otherwise my wound would not heal fast” (MP1).*

“*The nurses told me that I should not drink alcohol otherwise I would get drunk and fall and probably hit the sore on the ground or sit on the scrotum. I should avoid taking cola nut since it can cause irritation that can make me cough and caused pains*” (MP7).

Some participants reported that the nurses told them that they should not lift heavy objects or do any heavy work that could lead to complication:

*“The nurse told me I should not lift any heavy object and I should not do difficult work that can cause me problem”* (FP4).

Some participants reported that nurses educated them to eat nutritious food when they were discharged.

*“The nurses told me I should be eating fruits, vegetables, oranges, fish, meat and others” (*MP3).

*“The nurses advised me that I should not eat hard food but I should be taking light soup, porridge to enhance easy defecation” (*FP6).

However, some participants stated that the nurses did not educate them on what food to eat but told them to come for their wound dressing.

*“No nurse discussed anything about nutrition to me, like I should eat this food or that food. It is only the dressing day they told to come back for my wound to be dressed”* (MP5).

### Education on medications

The nurses educated participants on how to take their medications at home prior to discharge but some did not indicate the time interval for the medication to be taken*:*

*“One nurses showed me that I should take one tablet in the morning, afternoon and in evening. She also told my sister to always remind me to take the medicine at home but she did not state the time interval for the medication to be taken”* (FP4).

*“The nurses showed me how I should take the medications at home. They told me that I should take the drugs in the morning, afternoon and evening without stating the specific hour I should take the medication”* (FP13).

Others were told to take the drugs regularly but not given specific details:

*“The nurses said I should be taking the medications regularly but they did not tell me the time I should take the drug”* (FP12).

Some participants also followed the inscriptions on the sachet to take their medications at home without prior education from the nurses.

*“The medications that they gave me from the hospital, it has been indicated on the sachet how it should be taken so I have been following that inscription to take my medications at home”* (FP15).

However, all the participants said the nurses did not educate them on the actions and side effect of their medications:

*“No nurse told me about the actions and side effects of the medications that I was taking”* (MP5).

*“The nurses did not educate me on the actions and the side effects of the medications I was taking at home”* (FP13).

### Education on wound care

All participants reported that the nurses told them to come to the ward for the daily dressing of their wounds. Participants were given different dates to come for the dressing. A participant stated*:*

*“The nurses told me to come to the ward every three days for the dressing”* (MP3). *“The nurse told me to come to the hospital every two days for the dressing”* (MP1).

All the participants reported that they received information from nurses on things that they should do to prevent wound infection.

*“The nurses told me that I should not let water get to the wound site to promote infection”* (MP3).

*“My wife always cleaned my body with sponge, soap and water because the nurses told me that the wound should not be wet”* (MP5).

However, some participants said they were not told to keep the wound dry:

*“The nurses did not tell me that I should keep my wound dry” (*FM14).

Some participants added that nurses advised them to keep the wound clean:

*“…She said I should not expose the wound to dirty things like my dress and I should wash my clothing well”* (MP1).

Other participants stressed that nurses did not educate them on the early detection of the signs of wound infection for them to report early:

*“The nurses did not educate me on the signs of wound infection to enable me detect the infection early and report to the hospital”* (FP4).

Some participants reported that some of the nurses would dress their wounds and express the pus whilst others did not do so:

*“Some nurses would dress the wound and pressed it for all the pus to come out. But other nurses would dress the wound without pressing and you would not even feel any pain”* (MP5).

However, all the participants reported that the nurses did not educate them on the signs of wound infection:

*“The nurses did not teach me the signs of wound infection” (FM10). “The nurses did not tell me any danger signs of wound infection”* (MP8).

### Follow up/ hand over patients to community health nurse

The participants reported that no nurse visited them at home or even called them on phone to find out how they were doing at home. A participant stated that some nurses informed her to come for review but if there was a problem, she should report to the hospital immediately:

*“Some nurses said I should take note of the day that they told me to come for review but if there is a problem I should come and should not wait for that day. They did not visit me at home”* (MP9).

*“The nurses never visited me at home following my discharge from the ward but rather, theytold me that I should come back today for them to see how I was doing at home.”* (FP14).

*“I wish nurses had visited me at home to see how I was doing. I believed my wound got infected at home*. I*f a nurse had visited me at home she could have prevented this”* (MP3).

All the participants stated that nurses did not hand them over to the community health nurse to continue with treatment.

“*Nurses did not visit me at home and they did not hand me over to the community health nurse to continue treatment”.*

“*Hmmm I was thinking that the nurses will tell me to go to the community health nurse for my wound dressing but they did not and I did not also go to her”.*

### Nurse-patient interaction

Some participants added that some nurses were good and polite any time they came for dressing;

*“In fact some of the nurses were good. When they come in the morning they would greet me nicely and chat with me. Some were also showing respect to me by speaking politely to me and some would ask me whether I had any problem that I want to share with them”* (MP3).

Some participants also stated that the nurses have been taking care of their wound well for them to recover fast.

*“I am so happy that I have finally done the operation and dressing is also good. At first I could not work effectively because of my swollen scrotum but now I do the basic work without any problem”* (MP7).

“*As for the dressing, the nurses in the room are doing well. In fact they dress my wound very well. Look at the wound it is almost healed after two weeks of operation”* (MP1).

However, some participants felt that the nurses did not communicate well with them during outpatient wound dressing.

*“My wound gaped and I was rushed to the ward and the nurses were shouting at me saying that I was careless”* (FP4).

A few of the participants said nurses spoke rudely to them:

*“One nurse spoke rudely to me at the dressing room. She said you cannot put medicine down and it poured. I could not talk and my tears were flowing from my eyes.”* (MP8).

Some participants also stated that some nurses deliberately refused to dress their wounds with the excuse that they (participants) came late.

*“The nurses refused to dress my wound saying that I came late even though I was feeling pain at the site of the wound. They only added a plaster to hold the old dressing in place”* (MP3).

As a result of these attitudes of some nurses, participants had these pieces of advice.

*“Hmm, if they could train nurses to dress wound nicely it would help to reduce infection. The doctors would do the operation nicely and everything would be fine but because nurses do not dress the wounds well, the wounds are always infected”* (MP5).

Some participants pleaded that nurses should talk nicely to participants to allay their anxiety:

*“It is good that nurses should talk nicely to patients during any procedure they are doing especially those who have never had an operation so that their anxiety and fear will be better”* (FP14).

## Discussion

The study identified that nurses advised patients to stop certain lifestyle practices such as taking alcohol and smoking cigarette after discharge in order to promote their speedy recovery at home. Besides, nurses educated some patients on the need to keep their wounds dry to prevent infection. These findings support previous studies that indicated that nurses often planned with patients and their family members during discharge and educate them to avoid factors that can negatively affect recovery. They also educate patients on proper personal hygiene and prevention of wound complications [1, 3].

Proper nutrition promotes speedy recovery and enhances wound healing [8]. However, the study indicated that nurses did not stress the importance of nutrition to some patients even though some were encouraged to take fruits, vegetables, fish and meat upon discharge to promote wound healing. It could be inferred from the study that nurses did rush to educate some patients and thereby providing them with incomplete information or information that they could not understand [6].

Educating discharged patients on how to take their prescribed medications at home to enhance speedy recovery is important since failure to do so could lead to patients taking overdose or under dose of such medicines [8]. Besides, it is important to educate patients on the side effects of these medicines [9, 10]. However, the study revealed that some patients were not educated on how to take the medicine at home especially on the prescribed time intervals and this compelled some of them to follow instructions on the label. Besides, patients were not educated on the actions and the side effects of these medications. It is good nurses and pharmacists take their time to educate discharged patients on their prescription instructions to avoid inappropriate usage of medicine at home.

The study revealed that patients were informed of their scheduled days for wound dressing on the ward without educating them on how to change their wound dressing when necessary. Besides, patients were not educated on some of the signs of wound infection. These findings confirm previous studies that indicate that ineffective teaching of surgical patients upon discharge about wound care could lead to patients lacking the requisite knowledge and skills on how to care for the wound at home leading to complications [4, 5, 7]. It is therefore mandatory that nurses spend much time in educating discharged surgical patients to equip them with the necessary knowledge and skills to enable them handle their wounds properly at home. Besides**,**the study found that some nurses refused to dress some patients’ wounds during follow up visit to the hospital dressing room and such patients perceived nursing care as poor. These practices compounded some the patients’ problems contributing to their wound infection.

Nursing is a continuous process and there is the need to visit discharge patients at home to find out how they are doing. However, nurses at the surgical unit did not visit patients at home. Many of the patients stated that some nurses told them their wounds were infected when they reported to the ward for dressing. This confirms previous studies that indicate that due to the untimely and infrequent or lack of follow up visits that nurses do, many patients get wound infection at home. Wound infection puts much burden on family members who lack the requisite knowledge to handle it leading to more complications [7, 15, 16]. To close this gap in the nursing care, it is suggested that nurses should incorporate days in their duty schedule to visit discharged patients to assess their environment and provide education as necessary [13].Also, nurses could liaise with the community health nurses to continue caring for patients at home [18] since failure to do so could affect recovery and cause readmission [31].

In surgery, patients’ perspectives about care are relevant as a key outcome in areas such as health related quality of life and satisfaction. Hence, some patients were happy and thankful to some nurses for being polite in their communication and interaction during care confirming previous studies that show that good nurse-patient interaction and communication are the key component of quality care since it establishes a healthy relationship that allows participants to voice their concerns freely [8, 26]. However, the study also found that some nurses were using abusive language for patients and were not ready to listen to their concerns as stated in previous studies [27].

However, some patients were full of praises that some nurses were able to dress their wounds skilfully as supported by a previous study [21]. It was also reported that some patients were happy to have resumed their activities of daily living which is supported by previous studies that show that patients desire to get good outcomes such as regaining their normal activities [19, 20].

## Conclusion

In conclusion, the study explored the experiences of discharged surgical patients on their preparation by nurses toward home management and how they manage their condition at home either by themselves or by the community health nurses and as outpatients at the dressing unit at regional hospital, Bolgatanga. Despite the fact that nurses did their best to ensure discharged patients were able to manage their conditions at home, the findings from the study indicated that nurses never visited patients at home and never handed the discharged patients over to the community health nurses to continue management. Nurses also exhibited poor attitude such as poor communication toward patients during outpatient care.

The relevance of the study was to find out the challenges that discharged patients faced following the management of their condition at home and the way forward. From the study findings, it is importance to note that nurses at the surgical unit do not adequately prepare patients towards discharge leading to a serious gap on how they should handle themselves well to prevent infections. It therefore important for the hospital management to organize refresher training on patient discharge preparation, nurse-patient interaction, home visit and the need to hand over patients to community health nurses to continue treatment at home. Even though, the study specifically explored the perspectives of surgical patients on nurses’ education following discharge, some discharged patients did indicate that family members play a major role intheir home management. Hence, it is recommended that further studies should be conducted on the impact of family members on discharged patient home management.
